# Current state of the global impact of cannabis use

**DOI:** 10.47626/2237-6089-2022-0002

**Published:** 2022-07-29

**Authors:** Thiago M. Fidalgo, Lisia von Diemen

**Affiliations:** 1 Departamento de Psiquiatria Escola Paulista de Medicina Universidade Federal de São Paulo São Paulo SP Brazil Departamento de Psiquiatria, Escola Paulista de Medicina da Universidade Federal de São Paulo (EPM-UNIFESP), São Paulo, SP, Brazil.; 2 Programa Jovens Lideranças Médicas Academia Nacional de Medicina Rio de Janeiro RJ Brazil Programa Jovens Lideranças Médicas, Academia Nacional de Medicina, Rio de Janeiro, RJ, Brazil.; 3 Departamento de Psiquiatria e Medicina Legal Universidade Federal do Rio Grande do Sul Porto Alegre RS Brazil Departamento de Psiquiatria e Medicina Legal, Universidade Federal do Rio Grande do Sul (UFRGS), Porto Alegre, RS, Brazil.; 4 Hospital de Clínicas de Porto Alegre Porto Alegre RS Brazil Coordenação de Saúde Mental, Hospital de Clínicas de Porto Alegre (HCPA), Porto Alegre, RS, Brazil.

Marijuana is the illicit drug most used worldwide, with more than 200 million people using it worldwide in 2019,^1^ representing around 4% of the global population. This alone would be a solid argument to justify the producing this special issue of *Trends in Psychiatry and Psychotherapy*. All healthcare providers, not only psychiatrists, should have access to high-quality material on this topic to better advise patients and their families about the potential risks and benefits of its use. However, the past few years have seen an increase in the literature concerning cannabis use, covering a myriad of consequences of this behavior, ranging from general and mental health impairments to potential medicinal applications; from legal and criminal implications to a possible explanation for mass incarceration; from the cause of social deprivation to a consequence of social vulnerability. These potential paradoxical facets and the high speed at which literature is published make it difficult for professionals in the area to keep updated.

Therefore, this partnership between the World Psychiatric Association (WPA) and *Trends in Psychiatry and Psychotherapy* is admirable. And it was a great honor for us to act as guest editors of this supplement titled “Current state of the global impact of cannabis use.” Our first challenge was to establish the scope we would cover. Among the many possible editorial lines that could be followed, we decided to include manuscripts discussing cannabis dependence prevention, treatment, and related topics. This decision was based mainly on the audience that *Trends* impacts and on the general scope of the journal. Although extremely rich and vast, articles on medicinal uses of cannabis were considered beyond the scope of this supplement and the subject should deserve a whole issue dedicated to discussing it.

This exciting supplement features two original articles, one addressing the role of friends in young cannabis users^2^ and the other, the clinical correlates of impulsivity in cannabis use disorder.^3^ Along with this, we have two reviews covering critical aspects of the world’s epidemiology, policies, legalization, and outcomes of the non-medical use of cannabis.^4,5^ Two editorials from leading experts in the field round out this issue, exploring distinct facets of cannabis legalization and its outcomes.^6,7^

We thank the authors from different countries who submitted their manuscripts to this issue, the reviewers who made the peer review assessment of the manuscripts, and the authors of the invited editorials. We also thank the full support of the editorial team of *Trends* and the WPA. We hope this joint effort will be a step further in such a vibrant and transforming field.
